# Evaluation of Maternal Mortality Cases in the Province of Elazig, Turkey, 2007-2013: A Retrospective Study

**DOI:** 10.5539/gjhs.v7n1p188

**Published:** 2014-08-31

**Authors:** Salih Burcin Kavak, Ebru Celik Kavak, Ismail Demirel, Abdurrahim Turkoglu, Ibrahim Halil Akkus, Rasit Ilhan, Selcuk Kaplan

**Affiliations:** 1 Fırat University, School of Medicine, Fırat Medical Center, Department of Obstetrics and Gynecology, Elazıg, Turkey; 2 Special Medical Park Hospital, Department of Obstetrics and Gynecology Elazıg, Turkey; 3 Fırat University, School of Medicine, Fırat Medical Center, Department of Anesthesiology and Reanimation, Elazıg, Turkey; 4 Fırat University, School of Medicine, Fırat Medical Center, Department of Forensic Medicine, Elazıg, Turkey; 5 Elazıg Public Health Directorate, Department of Family Medicine, Elazıg, Turkey

**Keywords:** mortality, delay models, causes of death

## Abstract

The aim of this study was to determine the causes and factors influencing maternal mortality.

All maternal deaths occurring between January 2007 and November 2013 in the Elazıg Province of Turkey were retrospectively investigated. The maternal age, obstetric history, cause of death, encountered delay model of each case, as well as the overall number of annual live births in the Province were determined. The information of cases was obtained from Directorate of Public Health and hospital records. Families or family doctors were also interviewed to obtain details about the circumstances surrounding each death. There were a total of 64,423 live births in the Province of Elazıg between 2007–2013. The number and ratio of maternal deaths due to direct and indirect causes were 12 and 18.6, respectively. The direct causes of maternal death were hypertensive diseases of pregnancy (n=5, 41.7%), obstetric hemorrhages (n=3, 25%) and pulmonary embolism (n=1, 8.3%). The indirect causes of death were cardiac diseases (n=2, 16.7%) and malignancy (n=1, 8.3%). When classified according to the “Three Delays Model”, 2 cases were in the first delay model and 3 cases in the third delay model; the second delay model led to no maternal deaths.

Hypertensive diseases of pregnancy are the leading cause of maternal mortality in our province. The preventable causes of maternal mortality and factors contributing to death must be identified to reduce the incidence.

## 1. Introduction

Maternal mortality remains a common public health problem, especially in the developing countries (Brown et al., 2013; United Nations, 2010). Relatively few women die in the perinatal period in countries with high income. The maternal mortality ratio (MMR) is the annual number of female deaths per 100,000 live births from any cause related to or aggravated by pregnancy and its management or within 42 days of termination of pregnancy (excluding accidental or incidental causes). MMR reflects the quality of obstetric care, and can be used for international comparisons (Schutte et al., 2010).

In developed countries, maternal mortality ratios have been stabilized at 5–10 (Chuitemaker et al., 1997). Of the approximately 273,000 maternal deaths that occurred worldwide in 2011, nearly all were in developing countries, and more than half in Sub-Saharan Africa (Lozano et al., 2011).

Identification of the causes of maternal deaths and delays that contribute to death are important in planning health facilities.

Among the Millenium Development Goals of United Nations three-quarter reduction of maternal mortality by 2015 is aimed (United Nations, 2000).

The purpose of our study is to identify the causes and delay models of maternal mortalities in an area of Turkey with low income.

## 2. Method

In this study, the maternal deaths occurring between January 2007 and November 2013 at health centers of the Ministry of Health Department in the Elazıg Province of Turkey were retrospectively investigated. For this purpose, the data on the “Maternal Death Record Forms” of the Health Department were studied. Additional information was obtained from interviews with family members, family doctors and the Forensic Medicine Institude. The study was approved by the Local Ethics Committee. The age, gravidity, and parity of each case, direct and indirect maternal deaths, accidental maternal deaths, and year of death were noted. Based on the total live births and maternal deaths in 7 years, maternal mortality ratio was determined. For the evaluation of the data obtained, the SPSS 21.0 Statistical Program was used, and descriptive statistics were performed.

## 3. Results

There were a total of 19 maternal deaths in the Province of Elazıg between January 2007 and November 2013. During these 7 years, the total number of live births was 64,423. The ratio of maternal mortality based on direct and indirect causes of death was calculated as 18.6. This ratio excluded accidental causes. The distributions of causes of maternal mortality are shown in Table 1.

**Table 1:** The distributions of causes of maternal mortality in the province of Elazig between 2007-2013

Year	Number of Live Births (Number)	Mortality During Pregnancy (Number)	Postpartum Death (Number)	Direct and Indirect Causes of Death (Number)	Accidental Cause of Death (Number)	Total Deaths (Number)	Maternal Mortality Rate(/100000)
***2007***	8529	1	2	3	-	3	35,2
***2008***	9493	2	0	1	1	2	10,5
***2009***	9449	0	1	-	1	1	-
***2010***	9447	4	3	5	2	7	52,9
***2011***	9761	-	-	-	-	-	-
***2012***	9853	-	-	-	-	-	-
***2013 (The first ten months)***	7891	3	3	3	3	6	38,0
***Total***	**64423**	10	9	**12**	7	19	**18,6**

The average of maternal age, gravidity, and parity were identified as 32.6±8.9 (range 17–46), 4.0±2.4 (range 1–8), and 2±15 (range 0-5), respectively.

Of the 19 deaths, 12 (63.1%) were due to direct and indirect causes and 7 (36.9%) were accidental maternal deaths. Ten mother died during the pregnancy period and 9 died after delivery.

The most common direct cause of maternal mortality was the hypertensive disorders of pregnancy (41.7%, n=5) followed by obstetric hemorrhages (25%, n=3) and pulmonary embolism (8.3%, n=1). The indirect causes of death were cardiac diseases (16.7%, n=2) and malignant melanoma (8.3%, n=1).

Of all deaths, the most frequent cause of accidental maternal deaths were traffic accidents (15.8%), followed by earthquake (10.6%) and gunshot wounds (5.2%) but these were not included in the study. The distributions of causes of all maternal deaths are given in Table 2.

**Table 2:** Distributions of causes of all maternal deaths in the Province of Elazig between 2007-2013

Cause of Death	N (Number)	%		Ratio in Maternal Mortality (%)
***Direct Causes of Death***				
Hypertensive Disorders of Pregnancy	**5**	**26,4**		**41,7**
Obstetric Hemorrhage	**3**	**15,8**		**25**
Pulmonary Embolism	**1**	**5,2**		**8,3**
***Indirect Causes of Death***				
Cardiac Disease	**2**	**10,6**		**16,7**
Malignant Disease	**1**	**5,2**		**8,3**
**Accidental Causes of Death**				
Traffic Accident	**3**	**15,8**		**-**
Earthquake	**2**	**10,6**		**-**
After the injury of firearm	**1**	**5,2**		**-**
Cause of Death Undetermined	**1**	**5,2**		**-**
**Total**	**19**		**100**	**100**

When classified according to the “Three Delays Model” proposed by the World Health Organization (WHO), 2 cases were in the first delay and 3 cases in the third delay model. There were no maternal deaths in the second delay model. There were no delays in 7 cases. Also, there were no delays in accidental deaths (n=7). The maternal mortality delay models are shown in Table 3.

**Table 3:** The delay models of maternal mortality between 2007-2013 in the province of Elazig

Year	Maternal Death (Number)	1. Delay Model (Number)	2. Delay Model (Number)	3. Delay Model (Number)	No Delay Model (Number)
**2007**	3	1	-	1	1
**2008**	2	1	-	-	1
**2009**	1	-	-	-	1
**2010**	7	-	-	-	7
**2011**	-	-	-	-	-
**2012**	-	-	-	-	-
**2013**	6	-	-	2	4
***Total***	***19***	***2***	***-***	***3***	***14***

## 4. Discussion

Although 99% of maternal deaths occur in underdeveloped countries, young and fertile women in developed countries also die from pregnancy-associated complications. According to the WHO the rates of maternal mortality are 55% in Asia, 40% in Africa, and only 1% in developed countries (World Health Organization, 2005).

The ratio of maternal mortality in Turkey used to be determined by discrete investigations carried out at certain times until the introduction of a National Maternal Mortality Monitoring Program in 2007. According to these investigations, the maternal mortality ratio based on every 100.000 live births was 20.8 in 1975 and 28.5 in 2005 (HUME Institute, 2006).

The “Maternal Mortality Data System” included in the Maternal Mortality Monitoring Program has been used since 2007. The aim of this program is to determine the causes of all maternal deaths and delay models and prevent avoidable mortality with the ultimate goal of reducing maternal mortality ratio under 15 by the end of 2010 and under 10 by 2014. Following the application of the Maternal Mortality Monitoring Program, the ratios determined were 21.3 in 2007, 19.4 in 2008, 18.4 in 2009, 16.4 in 2010, and 15.5 in 2011 (Turkish Ministry of Health, 2011).

Obstetric hemorrhages are still the primary cause of maternal death in underdeveloped and developing countries, particularly in rural areas where healthcare services are inadequate. Fatal hemorrhages are caused by postpartum atony, placental abruption, placenta previa, and less frequently by laceration of the cervix-vagina, abnormally located placenta, and uterus rupture (Fox et al., 1995). In our study, obstetric hemorrhages comprised 25% of maternal deaths whereas the main cause was the hypertensive diseases of pregnancy compromising 41.7%. It has been reported that in general the hypertensive diseases of pregnancy are the foremost cause of maternal deaths in Turkey (Aksu et al., 1998). Preeclampsia may develop at any time after 20 weeks of gestation. It is diagnosed when a pregnant woman develops blood pressure >140 systolic and/or >90 diastolic and 2+ proteinuria with dipstick in spot urine or 300 mg or more protein in a 24-hour urine sample. When convulsions are added to the disorder, it is then eclampsia. In case of preeclampsia-associated HELLP Syndrome (hemolysis, increased liver enzymes, low number of platelets), hemorrhages following liver failure and disseminated intravascular coagulation (DIC) may be life-threatening for the mother (ACOG, 2001).

In our study, 63.2% (n=12) of deaths were due to direct or indirect causes and 36.8% (n=7) were due to accidental causes. The most frequent direct cause of maternal mortality was the hypertensive disorders of pregnancy (41.7%, n=5) followed by obstetrical hemorrhages (25%, n=3) and pulmonary embolism (8.3%, n=1). The indirect causes were cardiac diseases (16.7%, n=2) and malignancy (8.3%, n=1). The ratio of maternal mortality directly and indirectly associated with pregnancy in the last 7 years was 18.6. The most frequent cause of accidental maternal death was traffic accidents, followed by earthquake and gunshot wounds.

It has been reported that maternal mortality and morbidity are associated with the advanced age of the mother, increased number of parity or abortions and low socio-economic status (Atrash et al., 1990). In our study, 50% of the cases were over 35 years of age and 25% were grand multiparous (more than 7 pregnancies). The causes of death in cases over 35 years of age (n=6) were postpartum hemorrhage in 3, cardiac disease in 1, eclampsia in 1, and malignancy in 1 patient. Two of three grand multiparous women died from postpartum hemorrhage and one of these died from cardiac disease.

According to the National Survey of Maternal Mortality report published by the Institute of Population Studies at Hacettepe University, this study conducted in 27 provinces in 2005, 18.4% of maternal deaths in Turkey were caused by preeclampsia/eclampsia. The ratio of pregnancy-associated maternal mortality was lowest in the Western Anatolian Region with 12.4 (±5.0) whereas it was highest in the North-Eastern Anatolian Region with 93.3 (± 17.2) (HUME Institute, 2006).

In the Province of Elazıg, the birth rates were higher than those in western regions. In the Middle-Eastern Anatolian Region where Elazıg is also located, the ratio of maternal mortality was 20.5. The results of our study demonstrated that the maternal mortality ratio in Elazıg was better than the relevant ratios found in other provinces of the region. According to the data published by the Statistics Institute of Turkey there were 1,237,172 live births in Turkey in 2011 where the rough birth rates were 15.7% in Istanbul, 16.2% in the Mid- Anatolia Region where the capital Ankara is located, and 22.4% in the Middle-Eastern Anatolia Region where Elazıg is located (Turkish Statistics Institute, 2011).

Maternal deaths are classified according to their causes and the delay models proposed by the World Health Organization (World Health Organization, 2004);

*Direct Maternal Death*s are those resulting from obstetric complications during pregnancy, labor or puerperium or resulting from any treatment received.

*Indirect Maternal Death*s are those resulting from a preexisting or newly developed disorder that were not due to direct obstetric causes but exacerbated by physiological effects of pregnancy.

*Accidental Death*s are deaths which occur during pregnancy, labor or ≥42 days after the end of pregnancy, which is directly or indirectly not related to pregnancy (For example; accidents, suicide, intoxications).

Evaluation of maternal deaths would take into account not merely the medical cause but also individual, community and health service factors that contributed to the deaths. Three Delays Model groups delays that causes maternal deaths into three as follows:

Delay Model 1: Delay in the deciding to seek healthcare; problems and poor understanding of complications and risk factors in pregnancy and inadequate social support.

Delay Model 2: Delay in reaching an appropriate medical facility; problems related to transportation, financial limitations, and limited number of healthcare centers.

Delay Model 3: Delay in receiving adequate care at health facility due to lack of medical supplies, medical staff or inadequately trained medical staff.

When the maternal deaths in our province are evaluated according to Three Delays Model, it is found that in 73.7% maternal deaths no delay was present. 10.5% were in Delay Model 1 and 15.8% were in Delay Model 3. Delay Model 2 was not encountered in Elazıg in the last 7 years.

Maternal mortality ratio was 22 between 2004 and 2008. This ratio dropped to 20 between years 2009 and 2013 (The World Bank, 2013). Hypertensive diseases are the most common cause of maternal mortality. Eclampsia is the most important reason for maternal mortality both in high and low income countries (Langer et al., 2008). Main cause of indirect maternal death has been Cardiac diseases are the main cause of indirect maternal deaths (Engin-Üstün et al., 2012). Maternal deaths are still occurring due to preventable causes. Delays in the management of patients are still prominent. In conclusion, identification of the causes and delays in maternal deaths will give light to planning of future health facilities.
